# Elevated Plasma Melatonin Levels Are Correlated With the Non-motor Symptoms in Parkinson’s Disease: A Cross-Sectional Study

**DOI:** 10.3389/fnins.2020.00505

**Published:** 2020-05-19

**Authors:** Linyi Li, Zhenxiang Zhao, Jianjun Ma, Jinhua Zheng, Shen Huang, Shiyu Hu, Qi Gu, Siyuan Chen

**Affiliations:** ^1^Department of Neurology, Henan Provincial People’s Hospital, People’s Hospital of Zhengzhou University, People’s Hospital of Henan University, Zhengzhou, China; ^2^Department of Neurology, Henan Provincial People’s Hospital, People’s Hospital of Henan University, Zhengzhou, China

**Keywords:** Parkinson’s disease, melatonin, non-motor symptoms, cross-sectional study, circadian rhythm dysfunction

## Abstract

**Objective:**

Melatonin is the major hormone produced and secreted at night by the pineal gland into the cerebrospinal fluid (CSF) and circulation. The relationship between plasma melatonin levels and Parkinson’s disease is not clear. The aim of the current study was to assess plasma melatonin levels in Parkinson’s disease (PD) patients and to analysis the relationship between plasma melatonin levels and non-motor symptoms.

**Participants and Methods:**

In this cross-sectional study, we evaluated 61 patients with idiopathic PD [males *n* = 30 (49.2%), average age 62.4 years (range: 46–73 years)] and a total of 58 healthy volunteers [males *n* = 30 (51.7%), average age 64.3 years (range: 45–70 years)] who participated in the study. Plasma melatonin levels were measured using an enzyme-linked immunosorbent assay. The severity of disease in PD patients was scored by the Unified Parkinson’s Disease Rating Scale and the Hoehn and Yahr Staging scale. The quality of life in PD patients was assessed by the 39-item Parkinson’s Disease Questionnaire. The non-motor symptoms were assessed by the 14-item Hamilton Anxiety Rating Scale, the 24-item Hamilton Depression Rating Scale, the Parkinson Disease Sleep Scale, the Epworth Sleepiness Scale and the Non-Motor Symptoms Scale for PD.

**Results:**

Compared with the healthy controls, the plasma melatonin levels were significantly higher in PD patients (12.82 ± 4.85 vs. 19.40 ± 4.23, *P <* 0.001). Plasma melatonin levels were significantly associated with the levodopa equivalent daily dose (*r = −*0.262, *P <* 0.05, *n* = 61). Higher plasma melatonin concentrations were detected in the negative cardiovascular symptom group than in the cardiovascular symptom group (20.13 ± 3.74 vs. 16.93 ± 3.74, *P <* 0.05). Higher plasma melatonin concentrations were detected in the non-sleep-disorders group than in the sleep disorders group (22.12 ± 5.93 vs. 18.86 ± 3.66, *P* < 0.05). In addition, the plasma melatonin concentration was higher in the group without gastrointestinal dysfunction than in the gastrointestinal dysfunction group (21.71 ± 4.44 vs. 18.35 ± 3.74, *P* < 0.05).

**Conclusion:**

This study revealed that the plasma melatonin levels in PD patients were significantly higher than those in healthy controls. Non-motor symptoms that were significantly negatively correlated with plasma melatonin levels were cardiovascular symptoms, sleep disorders, and gastrointestinal dysfunction. Plasma melatonin levels have the closest relationship with sleep disorders. There was a correlation between plasma melatonin levels and sleep quality in patients with PD. The remaining non-motor symptoms were not related to plasma melatonin levels.

## Introduction

Parkinson’s disease (PD) is a complex neurodegenerative disease characterized by the loss of dopaminergic neurons in the pars compacta (SNpc), resulting in the classic motor manifestations in PD patients. The clinical diagnosis of PD mainly relies on the presence of motor symptoms as the disease progresses. It has been shown that heterogeneous non-motor symptoms might occur earlier than dyskinesias, which could contribute greatly to the early diagnosis of PD (Kalia and Lang, 2015). The classic motor characteristics of PD can be attributed to the loss of nigrostriatal dopaminergic cells, while various of non-motor symptoms reflect more complex causes, including mitochondrial dysfunction, oxidative stress, circadian rhythm dysfunction, neuroendocrine, and metabolic abnormalities (Shen et al., 2017). Circadian rhythm (CR) is an endogenous biological rhythm produced by the Suprachiasmatic Nucleus (SCN), which plays an important role in the regulation of most physiological processes such as sleep-wake cycle, daytime alertness, sleep quality and neurotransmitter release (Golombek and Rosenstein, 2010). Most PD patients have abnormal circadian rhythm in the course of disease, but the relationship between circadian rhythm dysfunction and PD has not been fully clarified (Musiek and Holtzman, 2016).

As an endogenous synchronous neurohormone secreted by the pineal gland, melatonin is not only one of the important transmission signals in circadian rhythm, which can stabilize and coordinate circadian rhythm, but also can play a neuroprotective role through the blood–brain barrier due to its lipophilic and hydrophilic characteristics (Pardridge and Mietus, 1980). Melatonin also regulates physiological processes such as the sleep-wake cycle, hormone secretion, core body temperature, cognitive performance, and mood. Exogenous melatonin also plays a role in the reduction and recovery of circadian rhythm (Videnovic et al., 2014; Trotti and Karroum, 2016). Studies have found that there is a certain relationship between the level of melatonin in CSF and the motor symptoms of PD (Leston et al., 2009). Neuropathology and related imaging studies have also confirmed the changes in melatonin levels and the decrease in hypothalamus volume in PD patients (Breen et al., 2016). In addition, melatonin can also regulate the expression of genes and proteins related to the apoptotic signaling pathway, inhibit the formation of α-synuclein toxic oligomers, α-synuclein fibrils and antioxidant stress (Onoa et al., 2011). Apart from this, studies have shown that melatonin also has a mutual regulatory effect with dopamine in the circadian cycle. During the day, melatonin level decreases and dopamine level increases, while at night, melatonin level increases and dopamine level decreases (Shen et al., 2017). Therefore, regulation of circadian rhythm dysfunction may become a new target for therapeutic intervention. Intervention of circadian rhythm dysfunction in PD patients at the early stage of disease may be a new therapeutic approach, which can delay disease progression and improve patients’ quality of life (Willison et al., 2013; Lauretti et al., 2016).

In the clinic, the determination of neuropeptides or hormones in various body fluids has better safety and convenience. Melatonin can be used as a potential biomarker reflecting the dysfunction of PD, providing new ideas for the early diagnosis and treatment of PD. Previous studies have not reached a consensus on melatonin levels in PD patients (Lin et al., 2014; Videnovic et al., 2014), and there is a lack of further detailed studies on melatonin levels, characteristics of PD patients, medication status and non-motor symptoms. In this study, a cross-sectional control study was conducted on PD patients and healthy controls with age and sex matching to further explore the correlation between plasma melatonin levels and non-motor symptoms in PD patients.

## Materials and Methods

### Participants

We enrolled 61 patients with PD from December 2018 to June 2019 at the clinic of the Henan provincial People’s Hospital, Henan Province, China. The diagnosis of PD was made according to the International Parkinson and movement disorder society (MDS) Clinical Diagnostic Criteria (Postuma et al., 2015). The exclusion criteria were as follows: (1) patients who were over 85 years or less than 45 years; (2) patients who might have been pregnant or breast feeding; (3) patients who had severe complications; and (4) patients who were using melatonin during the preceding 2 weeks or benzodiazepines or other hypnotics during the preceding 4 weeks and throughout the run-in period (Laudon et al., 2014).

Volunteer healthy controls, age- and gender-matched, were recruited during the same time from the health examination center. A total of 58 healthy volunteers participated in this study. None were taking any hormone treatment during the study. All subjects were made aware of the contents of the study, and a written informed consent document was obtained. The research protocol was approved by the Ethics Committee of Henan Provincial People’s Hospital of Medical Science.

### Clinical Characteristics

General clinical data such as patient name, gender, age, course of disease, past history, and medication status were collected. The current medication is converted into levodopa equivalent daily dose (LEDD) according to formula (Tomlinson et al., 2010). Motor symptoms were assessed by Part III of the Unified Parkinson’s Disease Rating Scale (UPDRS). Non-motor symptoms were assessed by the Pittsburgh Sleep Quality Index, the 14-item Hamilton Anxiety Rating Scale, the 24-item Hamilton Depression Rating Scale, the Parkinson Disease Sleep Scale, the Epworth Sleepiness Scale, and the Non-Motor Symptoms Scale. Hoehn and Yahr staging and the UPDRS were used to measure the severity of the disease. All scale scores were completed once in the patient’s off period.

### Blood Sample Collection and the Detection of Plasma Melatonin

All samples were taken in darkness or under a weak-intensity light (<50 lux), and the subjects were lying down in the bed after an overnight fasting of 12 h (Zhao et al., 2009; Alamdari et al., 2014). The serum samples were stored at −80°C until biochemical analyses. Blood samples were drawn for the analysis of melatonin levels in PD patients. Plasma levels of melatonin were measured using an enzyme-linked immunosorbent assay kit (cat no. E01M0005 for humans, Shanghai Blue Gene Biotech, Co. Ltd., Shanghai, China) with the following characteristics: sensitivity, 1.0 pg/ml; intra-assay coefficient of variation, < 9%; and interassay variation coefficient of variation, < 10%. All samples were analyzed in triplicate and averaged.

### Statistical Analysis

Data are expressed as the mean ± standard error of the mean (*SEM*). The *Kolmogorov–Smirnov test* was used to check the data for normality. The means of two continuous normally distributed variables were compared by an *independent-sample Student’s t-test*. The *Mann–Whitney U-test* and *Kruskal–Wallis test* were used to compare the means of two groups of variables that were not normally distributed. *Spearman’s (or Pearson’s) correlation analysis* was conducted according to whether the variables were normally distributed. Statistical analysis was performed using SPSS version 22.0 (IBM Corporation, Armonk, NY, United States) and GraphPad Prism 7 (GraphPad Software, Inc., San Diego, CA, United States). The *P-value <* 0.05 was considered statistically significant.

## Results

### Clinical Characteristics

Among the subjects included in the study, the average diagnosis age of the patients was 56.56 years, 49.2% were males, and the time since diagnosis was approximately 5.8 years. The MDS-UPDRS Part III score was 37.41 and the PDQ-39 score was 42.51 (Table 1).

**TABLE 1:** Clinical and quality of life characteristics of 61 participants in PD groups.

**Variable**	**Mean (SD)**
Male,%	49.2
Age of diagnosis,Y	56.56 (7.07)
Time since diagnosis,Y	5.80 (3.47)
LEDD,MG	574.52 (408.27)
MDS-UPDRS part III score	37.41 (15.32)
MOB	11.74 (9.32)
ADL	9.46 (5.23)
EMO	6.87 (5.68)
STI	3.97 (4.50)
SOC	0.95 (2.04)
COG	4.64 (3.33)
COM	2.39 (2.47)
BOD	2.49 (2.18)
Total PDQ-39 score	42.51 (25.09)

*LEDD, levodopa equivalent daily dose; MDS-UPDRS, movement disorders society-Unified Parkinson’s Disease Rating Scale; MOB, mobility; ADL, activities of daily living; EMO, emotional well-being; STI, stigma; SOC, social support; COG, cognitions; COM, communication; BOD, bodily discomfort; PDQ-39, Parkinson’s Disease Questionnaire.*

### Plasma Melatonin Concentrations in PD Patients and in Healthy Controls

**FIGURE 1:**
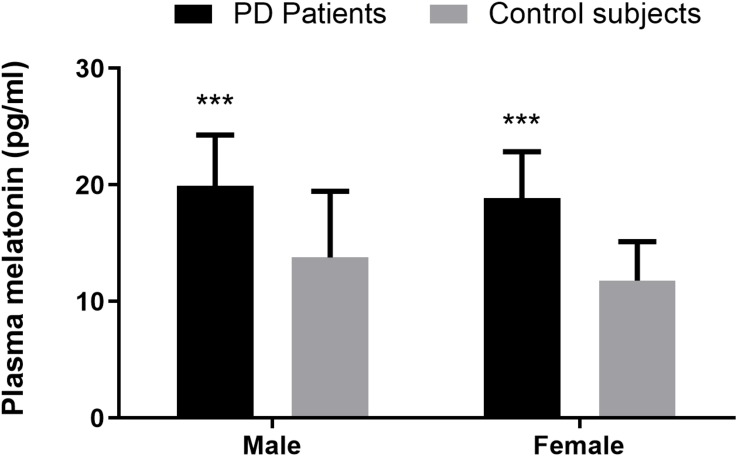
Plasma melatonin levels in PD patients and control subjects. Higher plasma melatonin concentrations were detected in PD patients than in control subjects. Values are presented as the *mean ± SEM*. ***P < 0.001.

Compared with the healthy controls, plasma melatonin concentrations were significantly higher in PD patients (19.40 ± 4.23 vs. 12.82 ± 4.85, *P* < 0.001). There were no significant differences on the basis of gender or age in plasma melatonin levels between PD patients and healthy controls (*P* > 0.05) (Figure 1).

### Correlation Between Plasma Melatonin Concentrations and Levodopa Equivalent Daily Dose

**FIGURE 2:**
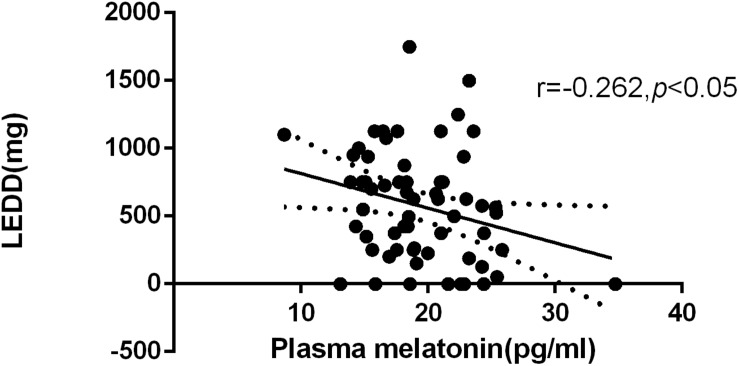
Relationship between plasma melatonin concentrations and levodopa equivalent daily dose. Plasma melatonin levels were negatively correlated with levodopa equivalent daily dose (*Pearson’s correlation coefficient r* = –0.262, *P* < 0.05, *n* = 61).

According to the formula for the LEDD, the average of LEDD was 574.52 mg. We found a negative correlation between plasma melatonin concentrations and LEDD (574.52 ± 408.27) (*Pearson’s correlation coefficient r* = −0.262, *P <* 0.05, *n* = 61) (Figure 2).

### Distribution of Plasma Melatonin Concentrations by Hoehn and Yahr (H & Y) Stage

**FIGURE 3:**
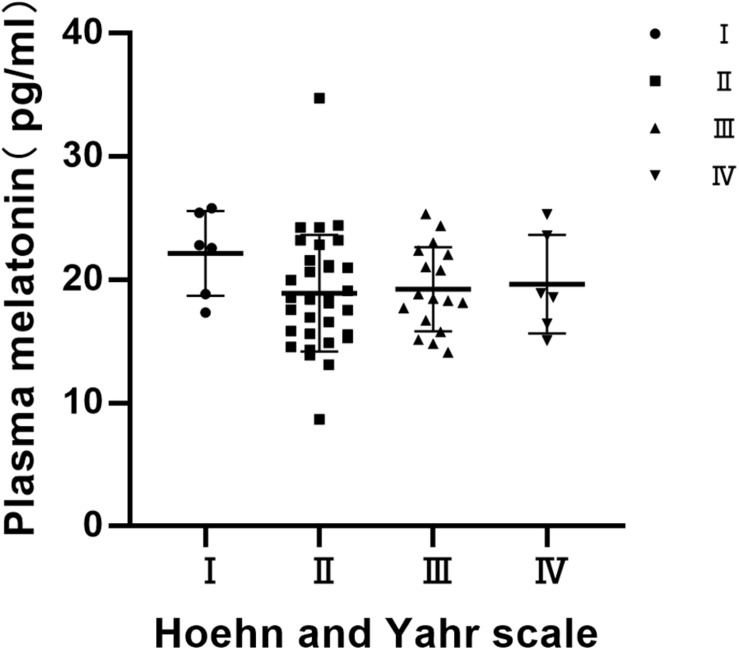
Distribution of plasma melatonin concentrations by Hoehn and Yahr (H & Y) stage. There were no significant differences in the distribution of plasma melatonin concentrations by Hoehn and Yahr stage in PD patients (*P* > 0.05).

Statistics on the Hoehn-Yahr classification of the PD group show that the Hoehn-Yahr distribution of the PD group is ranging from stage I to IV, and the median is 2. There were no significant differences in the distribution of plasma melatonin concentrations by Hoehn and Yahr stage in PD patients (*P* > 0.05) (Figure 3).

### Relationship Between Non-motor Symptoms and Plasma Melatonin Concentration in PD Patients

We performed a correlation analysis between non-motor symptoms and melatonin concentration, and we selected non-motor symptoms with a significant correlation and used them as a grouping criterion for Parkinson’s patients. The difference in melatonin concentration between the two groups was compared. This study included 61 patients with PD, including 14 with cardiovascular symptoms (23.0%), 42 with gastrointestinal dysfunction (68.9%), and 51 with sleep disorders People (83.6%).

This study found that the non-motor symptoms that were inversely related to plasma melatonin levels were cardiovascular symptoms (*Pearson/Spearman Rank* =−0.255, *P* < 0.05), sleep disorders (*Pearson/Spearman Rank* =−0.328, *P* < 0.05), and gastrointestinal dysfunction (*Pearson/Spearman Rank* =−0.265, *P* < 0.05). The strongest relationship was with sleep disorders. The remaining non-motor symptoms were not associated with plasma melatonin levels (*P* > 0.05) (Table 2).

**TABLE 2:** Correlation analysis of non-motor symptoms and plasma melatonin Levels in PD patients.

	**Mean (SD)**	**Pearson/Spearman rank**	***P*-value**
NMSS total score	51.69 (36.14)	–0.239	0.064
**NMSS component score**			
Cardiovascular	1.26 (2.84)	−0.255*	0.048
Sleep/fatigue	10.02 (8.30)	−0.328**	0.01
Mood/apathy	13.69 (13.59)	–0.102	0.436
Perceptual Problems/hallucinations	1.11 (2.37)	–0.204	0.115
Attention/memory	4.43 (4.16)	–0.08	0.542
Gastrointestinal tract	6.02 (6.44)	−0.265*	0.039
Urinary	6.8 (7.06)	–0.02	0.876
Sexual function	1.61 (3.01)	–0.067	0.608
Miscellaneous	6.75 (7.71)	–0.197	0.129

NMSS, The Non-Motor Symptoms Scale. Total scores are presented as *mean ± SD* and range. * P < 0.05, ** P < 0.01.

**FIGURE 4:**
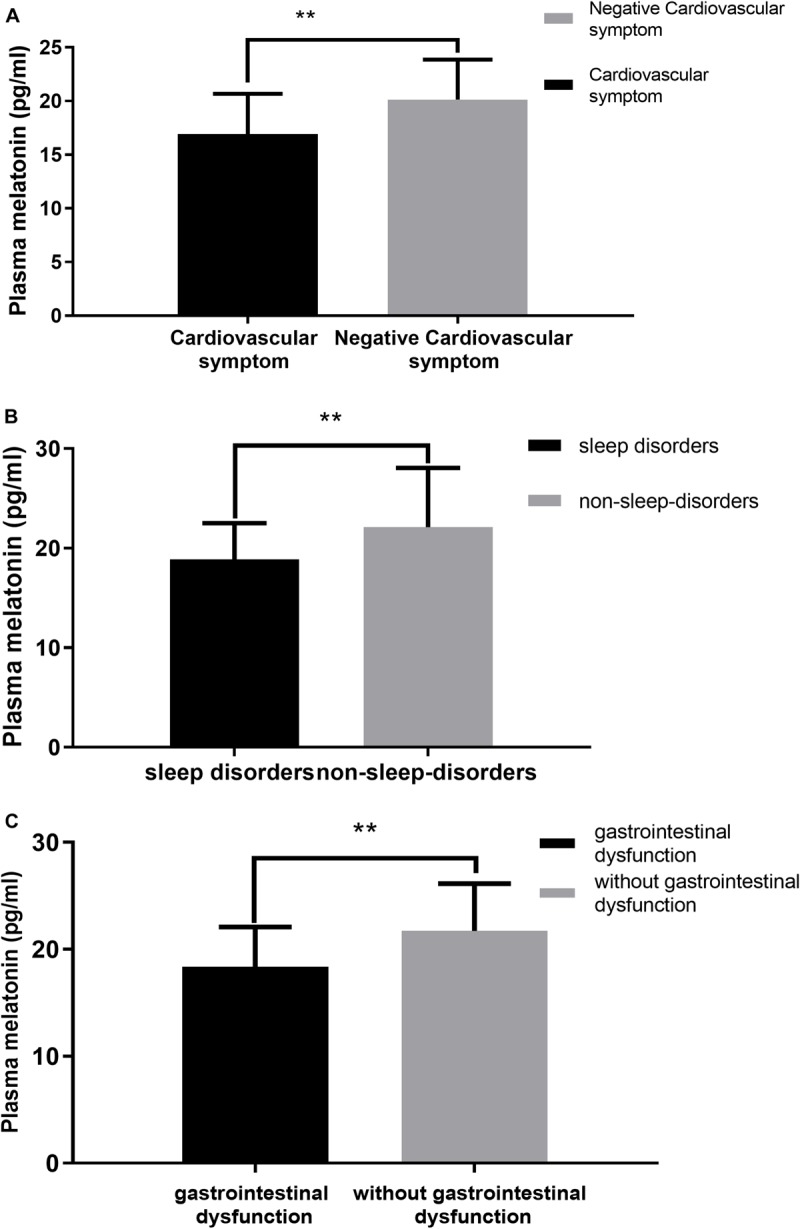
Relationship between plasma melatonin concentrations and non-motor symptoms. **(A)** Plasma melatonin levels in different subgroups of cardiovascular symptoms (with or without) in PD patients. Patients without cardiovascular symptoms showed higher levels of plasma melatonin. **(B)** Plasma melatonin levels in different subgroups of sleep disorders (with or without) in PD patients. Patients without sleep disorders showed higher levels of plasma melatonin. **(C)** Plasma melatonin levels in different subgroups of gastrointestinal dysfunction group (with or without) in PD patients. Patients without gastrointestinal dysfunction showed higher levels of plasma melatonin. Values are presented as the mean ± SEM. **P < 0.05.

Plasma melatonin concentration differences between the cardiovascular symptom group and the negative cardiovascular symptom group in PD patients. Higher plasma melatonin concentrations were detected in the negative cardiovascular symptom group than in the cardiovascular symptom group (20.13 ± 3.74 vs. 16.93 ± 3.74, *P* < 0.05) (Figure 4A). Plasma melatonin concentration differences between the sleep disorders group and the non-sleep-disorders group in PD patients. Higher plasma melatonin concentrations were detected in the non-sleep-disorders group than in the sleep disorders group (22.12 ± 5.93 vs. 18.86 ± 3.66, *P* < 0.05) (Figure 4B). In addition, the plasma melatonin concentration was higher in the group without gastrointestinal dysfunction than in the gastrointestinal dysfunction group (21.71 ± 4.44 vs. 18.35 ± 3.74, *P* < 0.05) (Figure 4C).

The values are presented as the mean and standard deviation (Figure 4).

### Relationship Between Plasma Melatonin Concentration and Sleep Quality in Patients With Parkinson’s Disease

To further investigate the relationship between plasma melatonin levels and sleep quality in PD patients, we performed a correlation analysis. We found that plasma melatonin levels in PD patients had a significant negative correlation with the PSQI total score (*Pearson/Spearman Rank* =−0.255, *P* < 0.05) as well as with the daytime dysfunction subitem (*Pearson/Spearman Rank* =−0.308, *P* < 0.05) and item 12 of the PDSS scale (*Pearson/Spearman Rank* =0.336, *P* < 0.05) (Table 3).

**TABLE 3:** Relationship between plasma melatonin concentration and sleep quality in patients with Parkinson’s disease.

	**Mean(SD)**	**Spearman rank**	***P*-value**
PSQI total score	8.05 (5.05)	−0.255*	0.047
**PSQI component score**			
Sleep latency	1.33 (1.20)	–0.112	0.389
Sleep duration	0.85 (1.04)	–0.144	0.269
Habitual sleep efficiency	1.26 (1.20)	–0.176	0.176
Sleep disturbance	1.34 (0.67)	–0.162	0.213
Use of sleeping medications	1.21 (0.94)	–0.036	0.782
Daytime dysfunction	0.31 (0.82)	−0.308*	0.016
Sleep quality	1.74 (1.08)	–0.179	0.167
PDSS total score	103.02 (24.06)	0.173	0.182
Item 1	6.36 (2.84)	0.062	0.634
Item 2	6.64 (3.71)	0.051	0.696
Item 3	6.10 (3.41)	0.071	0.585
Item 4	7.13 (2.95)	0.133	0.306
Item 5	6.95 (3.23)	0.173	0.181
Item 6	6.20 (2.86)	0.079	0.543
Item 7	8.92 (2.11)	0.177	0.173
Item 8	4.07 (3.37)	0.02	0.877
Item 9	8.34 (2.61)	0.052	0.689
Item 10	8.23 (2.63)	0.242	0.06
Item 11	8.18 (2.39)	–0.064	0.623
Item 12	8.33 (2.46)	0.336**	0.008
Item 13	5.92 (3.14)	–0.119	0.359
Item 14	6.41 (3.30)	0.15	0.249
Item 15	5.25 (3.21)	0.037	0.776
ESS total score	6.44 (4.69)	–0.079	0.545

ESS, Epworth Sleepiness Scale; PSQI, Pittsburgh Sleep Quality Index; PDSS, Parkinson’s Disease Sleep Scale. Total scores are presented as *mean ± SD* and range. * P < 0.05, ** P < 0.01.

## Discussion

More and more evidences show that PD patients have circadian rhythm dysfunction in both clinical symptoms and molecular level, and changes in circadian rhythm can also be observed in animal models of PD (Yuan et al., 2010). Melatonin is one of the biological indicators of endogenous output of circadian rhythm. The aim of this study was to analyze early-morning plasma melatonin levels and to further analyze the relationship between non-motor symptoms and melatonin levels in PD patients.

Consistent with the results of Catalá et al. (1997) and Lin et al. (2014), plasma melatonin levels were significantly higher in PD patients than in healthy controls. There were no significant differences on the basis of age or gender in plasma melatonin levels between PD patients and control subjects. The exact mechanism by which plasma melatonin is elevated in PD patients is unclear in this study. Since PD is characterized by irreversible degenerate dopaminergic neurons, it is possible that melatonin, as an endogenous signaling factor of circadian rhythm, may try to play a neuroprotective role and thus increase in compensation. Previous studies have demonstrated that endogenous melatonin levels increased with increasing dopamine degeneration in a 6-hydroxydopamine-induced rat model (Lin et al., 2013). In addition, we found that low levels of melatonin are associated with the progression of human cancer in previous studies and often indicate a higher risk of cancer. This may also be the reason for the low incidence of cancer in PD patients (Reiter et al., 2006). Since this study measured only the level of melatonin in the morning plasma, another possible explanation is that melatonin in the healthy control group had a regular circadian rhythm, whereas in Parkinson’s patients, circadian rhythm disorder caused an increase in morning melatonin reflexivity.

This study shows that patients with PD have a significant negative correlation with melatonin levels and levodopa equivalent doses, suggesting that the increased plasma melatonin level in PD patients may be related to the degeneration of dopaminergic neurons and the use of dopaminergic drugs. There is an interaction between the dopamine system and circadian rhythms. On the one hand, circadian genes regulate dopamine synthesis by regulating tyrosine hydroxylase. On the other hand, dopamine can regulate the expression of certain clock genes (Kawarai et al., 1997; Mcclung et al., 2005). Fertl, who found that compared to age-matched subjects, PD patients with melatonin secretion patterns evident in advance (Fertl et al., 1991), while the rhythm of this change may be due to central nervous system caused by levodopa therapy dopaminergic effects rather than the disease itself (Fertl et al., 1993). Studies on the circadian pattern of melatonin in PD patients have shown that dopaminergic treatment can significantly raise the level of plasma melatonin stage by stage, and the secretion of melatonin in PD patients with l-dopa-related motor complications increases during the day and significantly decreases at night. Studies have confirmed that dopamine plays an important role in maintaining the rhythmicity of the circadian system (Mendoza and Challet, 2014; Korshunov et al., 2017). Therefore, the increased melatonin level and the correlation with the equivalent dose of levodopa in PD patients may be related to the Circadian rhythm dysfunction of PD itself and the imbalance of the regulation of the dopaminergic system.

It has been revealed that the level of melatonin may be significantly associated with degenerative changes and disease severity in patients with PD (Lin et al., 2014). Unlike previous studies, in our study, plasma melatonin levels were not significantly associated with the severity of PD. Correlation analysis between the severity of the pathology of Lewy bodies in the SCN nucleus and the clinical circadian rhythm in PD has shown that the severity of the pathology of Lewy bodies in the SCN is not related to the overall neuropathological stage of the disease (Breen et al., 2014; De Pablo-Fernández et al., 2019). These all prove that the pathological changes of the SCN nucleus are independent of the pathological stage of Lewy bodies. Since the secretion of melatonin is based on the control of the SCN (Fifel, 2017), this may also be the reason why the plasma melatonin level of PD patients is independent of the severity of the disease.

This study classifies PD non-motor symptoms for the first time based on NMSS, and analyzes the correlation between non-motor symptoms and melatonin levels in PD patients. In the course of the disease, the plasma melatonin levels of PD and some non-motor symptoms such as cardiovascular symptoms, sleep disorders and gastrointestinal symptoms have a certain correlation, the most important being sleep disorders. The remaining non-motor symptoms have not yet been found to be relevant or different. Therefore, it is speculated that the occurrence of non-motor symptoms in PD patients has a certain relationship with internal circadian rhythm dysfunction.

The circadian rhythm system plays an important role in regulating the motor and non-motor symptoms and sleep disorders of PD. More than 60% of PD patients experience frequently relevant symptoms of sleep disturbances (Gómez-Esteban et al., 2010). Clinically, sleep disorders in PD patients often manifest as disturbances in sleep structure and changes in sleep time, mainly including excessive daytime sleepiness, insomnia, decreased total sleep time and sleep efficiency, and increased nighttime wakefulness (Belaid et al., 2015). Melatonin, which is mainly synthesized by the pineal gland, is known to play a key role in the circadian regulation of sleep and wakefulness (Garfinkel et al., 1997). It has been reported that in some PD patients (Van Gilst et al., 2013), a good night’s sleep can improve morning motor-related symptoms. On the other hand, higher morning melatonin levels can partly explain some common symptoms of PD patients, such as sleep disorders and daytime sleepiness (Atkinson et al., 2005). Our study found that plasma melatonin levels in PD patients have a significant negative correlation with the PSQI total score as well as with the daytime dysfunction subitem and item 12 of the PDSS scale was positively correlated with melatonin levels. Therefore, it can be speculated that the level of plasma melatonin in PD patients can reflect a certain degree of circadian rhythm dysfunction, and the circadian rhythm dysfunction may be one of the causes of daytime sleepiness.

Cardiovascular autonomic dysfunction is also a problem that cannot be ignored in patients with PD, and there is mounting evidence that melatonin has a pleiotropic effect in the cardiovascular system (Şehirli et al., 2013; Lochner et al., 2018). This study found that plasma melatonin levels were significantly higher in the asymptomatic group than in the cardiovascular group. Since melatonin has the effect of delaying atherosclerosis (Ma et al., 2018), anti-inflammatory and antioxidant (Mauriz et al., 2013), it is speculated that high levels of melatonin in PD patients without cardiovascular symptoms may reduce the activation of pro-inflammatory macrophages and the rupture of unstable plaques through these effects. Therefore, we can infer that endogenous melatonin may reduce cardiovascular symptoms by slowing the progression of atherosclerosis and reducing the incidence of cardiovascular adverse events to some extent.

Previous studies have shown that in rats, the gastrointestinal tract may be the largest source of exogenous melatonin in addition to the pineal gland. Melatonin can protect the gastrointestinal tract and regulate the duodenal mucosal barrier (Sommansson et al., 2013). The dysfunction of the brain-gut axis in PD patients may be related to the non-motor symptoms of gastrointestinal symptoms and also to the pathogenesis of PD itself (Felice et al., 2016). Studies have shown that patients with PD without gastrointestinal dysfunction have higher plasma melatonin levels. Considering the important role of the gastrointestinal system in PD, it is speculated that there is a certain relationship between the occurrence of gastrointestinal dysfunction and endogenous hormone circadian rhythm disorders in PD patients.

The study of circadian rhythms has opened up a new clinical and research approach, providing therapeutic targets for PD and neurodegenerative diseases. Chronic low-dose melatonin treatment can reduce the loss of dopaminergic neurons in the PD model and improve sleep disorders (Naskar et al., 2015; Carriere et al., 2016). In addition, the use of melatonin in patients with PD has been shown to help improve motor and non-motor symptoms, cognitive and emotional disorders (Bassani et al., 2014; Emre et al., 2014). At the same time, other therapies aimed at regulating circadian function, such as bright light therapy and exercise therapy, also have improve the non-motor symptoms such as sleep quality in patients with PD (Rutten et al., 2019). The above studies have confirmed that by affecting the regulation of circadian rhythm, melatonin has certain potential for the treatment of PD.

The limitations of this study include the small sample size, differences in study design methods, and melatonin assays that may be different from those of previous studies. Current research indicates that circadian rhythm dysfunction is associated with the clinical manifestations of PD, and changes in melatonin levels are associated with the non-motor symptoms in patients with PD. The relationship between melatonin and PD still needs further research, which is also valuable for exploring biomarkers for early diagnosis of PD.

## Conclusion

This study revealed that the plasma melatonin levels in PD patients were significantly higher than those in healthy controls. Melatonin levels were primarily associated with cardiovascular symptoms, gastrointestinal dysfunction, and sleep disorders. This finding suggests a potential underlying link between plasma melatonin levels and the non-motor symptoms in PD patients.

## Data Availability Statement

The datasets generated for this study are available on request to the corresponding author.

## Ethics Statement

The studies involving human participants were reviewed and approved by Ethics Committee of People’s Hospital of Zhengzhou University. The patients/participants provided their written informed consent to participate in this study.

## Author Contributions

LL and JM conceived and designed research. LL conducted experiments. ZZ, JZ, and SH performed data collection and statistical analysis. SYH, QG, and SC contributed data interpretation and literature search. LL and JM wrote the manuscript. All authors read and approved the manuscript.

## Conflict of Interest

The authors declare that the research was conducted in the absence of any commercial or financial relationships that could be construed as a potential conflict of interest.
